# Recognition of the mycobacterial cord factor by Mincle: relevance for granuloma formation and resistance to tuberculosis

**DOI:** 10.3389/fimmu.2013.00005

**Published:** 2013-01-24

**Authors:** Roland Lang

**Affiliations:** Institute of Clinical Microbiology, Immunology and Hygiene, University Hospital Erlangen, Friedrich-Alexander-University Erlangen-NürnbergErlangen, Germany

**Keywords:** mycobacteria, cord factor, TDM, Mincle, C-type lectin receptor, tuberculosis

## Abstract

The world's most successful intracellular bacterial pathogen, *Mycobacterium tuberculosis* (MTB), survives inside macrophages by blocking phagosome maturation and establishes chronic infection characterized by the formation of granulomas. Trehalose-6,6-dimycolate (TDM), the mycobacterial cord factor, is the most abundant cell wall lipid of virulent mycobacteria, is sufficient to cause granuloma formation, and has long been known to be a major virulence factor of MTB. Recently, TDM has been shown to activate the Syk-Card9 signaling pathway in macrophages through binding to the C-type lectin receptor Mincle. The Mincle-Card9 pathway is required for activation of macrophages by TDM *in vitro* and for granuloma formation *in vivo* following injection of TDM. Whether this pathway is also exploited by MTB to reprogram the macrophage into a comfortable niche has not been explored yet. Several recent studies have investigated the phenotype of Mincle-deficient mice in mycobacterial infection, yielding divergent results in terms of a role for Mincle in host resistance. Here, we review these studies, discuss possible reasons for discrepant results and highlight open questions in the role of Mincle and other C-type lectin receptors in the infection biology of MTB.

## TDM's history as PAMP and glycolipid effector molecule of mycobacteria

**Figure 1:**
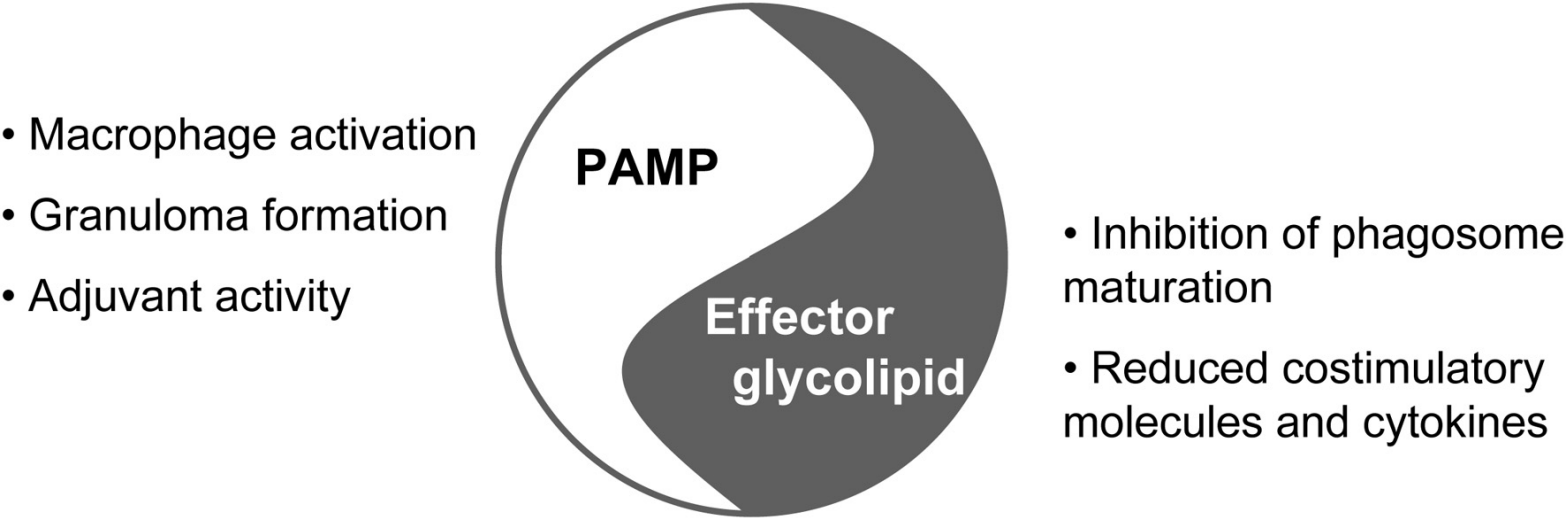
**TDM between PAMP and effector molecule**.

Trehalose-6,6-dimycolate (TDM) has been isolated as a major glycolipid from the cell wall of pathogenic mycobacteria in the 1950s by Bloch and colleagues (see Hunter et al., 2006 for review). The biological properties of TDM have been explored *in vivo* using mice and rabbit models and led to the realization that TDM is sufficient to induce formation of granulomas when injected in oil doplets (Yarkoni and Rapp, 1977; Ishikawa et al., 2009). These data indicated that TDM *per se* triggers an important reaction to mycobacterial infection, which has traditionally been viewed as a correlate of protective immune responses, required to wall off infection. TDM also possesses adjuvant properties and is contained in the experimental adjuvant Ribi (Gavin et al., 2006). While these properties of TDM indicated that it acted as a pathogen-associated molecular pattern (PAMP), indicating microbial danger and alerting innate immune cells, the cord factor has also been recognized since so long as a virulence factor of mycobacteria (Figure 1). First, extraction of glycolipids with petroleum ether from the cell wall of MTB rendered the mycobacteria avirulent, due to efficient killing of the TDM-less mycobacteria inside macrophages (Indrigo et al., 2002, 2003). Second, when replacing this rough and crippling procedure with genetic ablation of TDM biosynthesis, the attenuated phenotype was confirmed in ΔfbpA mutants lacking the mycolic acid transferase antigen 85A (Katti et al., 2008). In addition, these mutants triggered enhanced production of inflammatory cytokines and increased antigen presentation from infected macrophages (Kan-Sutton et al., 2009). Third, TDM coated onto beads is sufficient to delay phagosome maturation in macrophages (Axelrod et al., 2008), whereas the mutant MTB lacking TDM failed to block phagosome maturation (Katti et al., 2008). Together, the cord factor qualifies as a glycolipid effector molecule of pathogenic mycobacteria that reprograms macrophages for mycobacterial immune evasion and creation of a niche in the phagosome.

## TDM as PAMP

### A role for TLR?

Despite the decade-long knowledge of the inflammatory capacities of the cord factor, the mechanisms of immune cell activation by TDM were elusive until recently. As an abundant mycobacterial cell wall glycolipid with a chemical structure not found in vertebrate organisms, TDM *a priori* qualifies as a prototypical PAMP that may signal the presence of mycobacterial danger to innate immune cells. The phenocopying of inflammatory and granulomatous responses-induced by whole mycobacteria by TDM in animal models, and its long known property as an adjuvant eliciting cellular immune responses to co-administered protein antigen, support the notion of TDM being a PAMP. After the discovery of the toll-like receptor (TLR) family and several mycobacterial ligands that are recognized by TLR family members (ManLAM, 19 kDa lipopeptide), the search for a pattern recognition receptor sensing TDM naturally first focused on a potential role of TLR. In Geisel et al. (2005) reported that TDM-coated beads stimulated TNF and IL-6 production by macrophages *in vitro*, and that TDM in gel matrices injected s.c. into mice triggered massive leukocyte infiltration (Geisel et al., 2005). The role of TLR2 and TLR4, and of the adapter protein Myd88, was investigated exclusively for the *in vivo* leukocyte recruitment but not for the macrophage cytokine response *in vitro*. While TLR2^−/−^ and TLR4^−/−^ mice had no defect in leukocyte recruitment to TDM gel matrices, Myd88-deficient mice showed a severe reduction, pointing to a possible role for other TLRs or of other Myd88-utilizing receptors, e.g., for the cytokines IL-1 or IL-18. This initial report from the Russell lab was followed by a study reporting that murine macrophages deficient in TLR2 and in TLR4 had a strongly reduced response to TDM (Bowdish et al., 2009). In addition, the scavenger receptor MARCO was suggested to bind TDM in this study. Since scavenger receptors lack a cytoplasmic domain and are usually endocytic clearance receptors rather than activating PRR, a model was proposed where MARCO binding of TDM serves as a prerequisite for delivery to and activation of TLRs (Bowdish et al., 2009). In this context, a previous study is also of interest that investigated macrophages lacking Scavenger receptor A (SR-A) *in vitro* and observed an increased inflammatory response; importantly, these authors reported also that SR-A bound to the cord factor (Ozeki et al., 2006).

### TDM activates mincle-syk-card9 signaling

**Figure 2:**
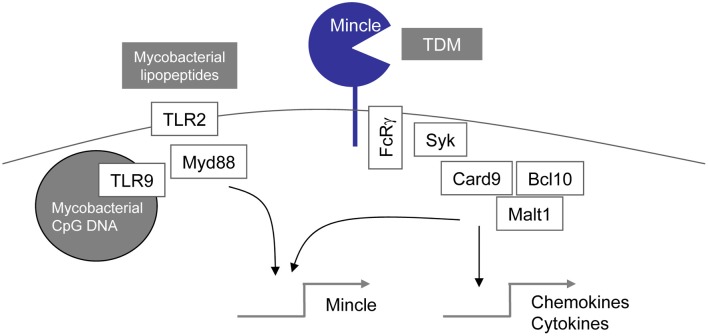
**TDM recognition and responses through regulated Mincle expression: possible synergy with TLR signals**.

Our own studies first aimed at determining a role of the TLR pathway for macrophage and DC activation-induced by TDM and its synthetic analog TDB. We observed that bone marrow derived macrophages from mice lacking Myd88 responded comparably to wild type cells (Werninghaus et al., 2009). In addition, the adjuvant activity of the cord factor analog TDB was preserved in mice deficient in TLR2, TLR3, TLR4, and TLR7 (Agger et al., 2008). This apparent lack of TLR-dependence led us to consider other PRR pathways. At this time, the C-type lectin receptor Dectin-1 (Brown, 2006) had just been described to activate macrophages via Syk-Card9 signaling (Gross et al., 2006). We employed macrophages from the respective KO mice to genetically define that macrophage activation by TDB and TDM requires Syk-Card9-Bcl10-Malt1 signaling, but is independent of Dectin-1 (Werninghaus et al., 2009). In contrast to Dectin-1 with its intracellular non-classical ITAM motif, other CLR activate Syk *via* association with an adapter protein carrying the classical ITAM motif (Robinson et al., 2006). The requirement for the adapter protein FcRg but not Dap12 pointed to a certain set of C-type lectin receptors, including the family member Mincle (gene symbol: Clec4e) that was expressed inducibly in macrophages stimulated with the glycolipids. Mincle had been described in 2008 by Yamasaki et al. as sensor of necrosis that binds SAP130, a splicing factor released from dying cells (Yamasaki et al., 2008). Using independent lines of Mincle KO mice, Yamasaki et al. and Wells et al. demonstrated a function of Mincle in the recognition of fungal carbohydrates in the cell wall of Candida and Malassezia yeast (Wells et al., 2008; Yamasaki et al., 2009). A role for Mincle in recognition of mycobacteria was suggested by Yamasaki's group through experiments using a Mincle-reporter cell line. In a series of elegant experiments, they then identified the TDM from cell wall lipid fractionations as the active compound, showed direct binding of Mincle to TDM, and demonstrated that Mincle^−/−^ mice fail to produce the granulomatous response to TDM observed in the lungs of wild-type mice (Ishikawa et al., 2009). In parallel, experiments with Mincle-deficient mice in our lab showed that TDB and TDM do not activate macrophages from these mice anymore and that the Th1/Th17 adjuvant effect of TDB requires Mincle *in vivo* (Schoenen et al., 2010). Together, these studies provided solid evidence that Mincle is an essential receptor for the mycobacterial cord factor (Matsunaga and Moody, 2009) (Figure 2). Until now, however, the requirement for Mincle in TDM recognition has only been shown in the mouse system, and the possibility that the response to the cord factor in human macrophages is mediated by the same receptor and pathway remains to be proven experimentally (Lang et al., 2011).

### Potential for synergy and cross-regulation of TLR and mincle signaling

The reasons for the discordant results concerning the involvement of TLR-Myd88 signaling for TDM recognition between the Bowdish et al. (2009) and our studies (Werninghaus et al., 2009; Schoenen et al., 2010) are at present unclear. Differences in the type of macrophage used (bone marrow derived vs. resident peritoneal macrophages), culture conditions, and the presentation of TDM (coated onto the cell culture dish vs. presented on beads) may be responsible. In general, TLR and CLR pathways can synergize to activate gene expression in macrophages. This has been clearly shown in the case of zymosan-triggered activation of macrophages through Dectin-1 and TLR2 (Dennehy et al., 2008, 2009). In the case of Mincle-mediated recognition of the cord factor, the inducible expression of the Mincle receptor following stimulation with TLR ligands (Matsumoto et al., 1999) suggests a mechanism of priming of macrophages for increased sensitivity to the CLR ligand TDM (Figure 2). The role of the MARCO-TLR2-Myd88 pathway described by Bowdish et al. for TDM recognition (Bowdish et al., 2009) may therefore consist in licensing of macrophages for TDM responsiveness through induction of Mincle expression. A synergistic effect of Myd88 and Mincle for TDM responsiveness has very recently been shown for neutrophils by Lee et al. (2012): Myd88^−/−^ neutrophils responded equally well with TNF release and adhesion to TDM as WT; in contrast, upon combined treatment with the TLR2 ligand Pam3CSK and TDM, synergistic induction was observed in WT but not Myd88^−/−^ neutrophils, creating a significantly reduced response in Myd88^−/−^ vs. wild-type neutrophils that correlated with a lack of up-regulation of Mincle in the Myd88^−/−^ cells (Lee et al., 2012).

## Is mincle required for anti-mycobacterial immunity? contradictory answers from mouse infection models

While the identification of Mincle as receptor for the cord factor solved a longstanding issue in TB research and provided a molecular basis for the adjuvant effect of TDM and TDB, the medically most important question remains whether Mincle and its associated pathway are required for anti-mycobacterial immunity, or may to the contrary be used by the pathogen to subvert immune responses.

### *In vitro* studies with phagocytes

The first steps taken to address the importance of the Mincle-Syk-Card9 pathway in the dealing of innate immune cells with infecting mycobacteria were *in vitro* infection experiments of macrophages with M. bovis *BCG* or MTB. These experiments showed a differential requirement for Mincle-signaling in the expression of several genes-induced by mycobacterial infection. Mincle-deficient macrophages expressed 10-fold less G-CSF and IL-6 in response to BCG (Schoenen et al., 2010). More recently, Behler et al. confirmed the essential role of Mincle for inflammatory cytokine and chemokine production in response to BCG in alveolar macrophages (Behler et al., 2012). Ishikawa et al. observed a slight reduction in the levels of several chemokines in Mincle^−/−^ macrophages infected with MTB; this dependence on Mincle was strongly enlarged when macrophages from a Myd88^−/−^ background were used (Ishikawa et al., 2009). Thus, a picture emerges that in the absence of Mincle macrophages can still sense mycobacteria through recognition of PAMPs other than TDM, but the inflammatory response is compromised at least for a subset of response genes. To date no published data is available that has tested whether phagocytosis of mycobacteria is reduced or delayed in Mincle^−/−^ macrophages.

### *In vivo* mycobacterial infection models

To date, three publications have reported on experimental mycobacterial infection in Mincle^−/−^ mice (Behler et al., 2012; Heitmann et al., 2012; Lee et al., 2012) (see Table 1). Heitmann et al. used aerosol infection with MTB H37Rv and comprehensively analysed bacterial burden in lung and spleen, as well as the inflammatory and T cell responses in the lungs and draining lymph nodes at three different time points after infection. In addition, histopathological analysis of the lungs for the extent of infiltration, granulomatous response and immunocytochemistry for iNOS expression was performed. None of these readouts showed a significant difference between Mincle^−/−^ and WT control mice, leading to the conclusion that Mincle is not essential for the host response and protection against TB infection in mice. Low-dose aerosol infection with MTB is arguably the best mouse model for human TB; however, there are examples of knockout mice where the deletion of a gene did not cause a phenotype in the low dose model, but mice showed higher bacterial burden when challenged with a higher dose or by a different route (Reiling et al., 2002). To test for this possibility, Heitmann et al. also challenged Mincle^−/−^ mice with a high dose of MTB H37Rv, again with no discernible difference in the bacterial load.

**Table 1:** **C-type lectin receptors in mycobacterial infection**.

	**Dectin-1**	**Mincle**	**Signr3**
Mycobacterial ligand	Unknown	TDM	ManLAM
Expression of CLR	Macrophages and DC	Inducible in macrophages and DC;	Up-regulated in alveolar macrophages during infection
		High constitutive expression in granulocytes	
Phenotype of knockout mouse in mycobacterial infection	Slightly reduced bacterial load in the lung;	Increased bacterial dissemination after BCG infection (Behler et al., 2012);	Increased mycobacterial load early in infection;
	Unchanged adaptive immune response	Increased pulmonary load after MTB Erdman (Lee et al., 2012);	Intact adaptive immune responses
		Unchanged resistance after MTB H37Rv (Heitmann et al., 2012)	
References	Marakalala et al. (2011)	Behler et al. (2012); Heitmann et al. (2012); Lee et al. (2012)	Tanne et al. (2009)

The second study reporting on the phenotype of Mincle^−/−^ mice in MTB is the paper by Lee et al., who focused their interest on the expression and function of Mincle in neutrophilic granulocytes during *in vivo* and *in vitro* encounter with TDM (Lee et al., 2012). Corroborating and extending the results obtained by Yamasaki's group (Ishikawa et al., 2009), they showed that Mincle^−/−^ mice fail to mount a granulomatous response to intravenously injected TDM and that Mincle expression on neutrophils is required for recruitment to TDM-coated beads injected subcutaneously. They further demonstrate that neutrophils attach to plate-bound TDM *in vitro* and respond in a Mincle-dependent manner with up-regulation of integrins and production of reactive oxygen species. These results are interesting because they draw attention to neutrophils which express higher levels of Mincle than macrophages and can immediately respond to TDM. Lee et al. also demonstrate that depletion of neutrophils with an anti-Ly6G antibody reduced the initial granulomatous response and the levels of IL-6 and MCP-1 in the lungs of TDM-injected mice. The analysis of MTB infection in the Mincle^−/−^ mice in the study by Lee et al. consists of one low-dose aerosol infection experiment where bacterial loads in the lungs were determined at two time-points after infection, showing a significant but moderate increase (0.5 log_10_). The increased mycobacterial numbers in the Mincle^−/−^ mice were paralleled by a stronger expression of inflammatory genes in the lungs. Given the expression and function of Mincle in granulocytes described in the same paper, it would be interesting to know whether neutrophil accumulation, integrin expression and cytokine production in the lungs is particularly affected in the mice infected in the Lee et al. study. The higher expression of inflammatory genes in Mincle^−/−^ lungs is difficult to interpret because it could be entirely due to the increased mycobacterial load generating more PAMPs for activation of receptors other than Mincle. One can only speculate at which time after infection Mincle^−/−^ mice start to have higher MTB burden in the lung; one possibility is that a lack of recruitment and proper activation of neutrophils early after infection gives MTB the ability to survive and grow better than in WT mice.

In contrast, the third study, reported by Behler et al., used the vaccine strain *M. bovis* BCG in intratracheal and intravenous infection models (Behler et al., 2012). These authors first characterized the expression of Mincle on alveolar macrophages following exposure to BCG *in vivo*, and observed a strong up-regulation of receptor levels on the cell surface. This is consistent with previous observations *in vitro* at the mRNA and protein level (Yamasaki et al., 2008; Werninghaus et al., 2009; Schoenen et al., 2010), and importantly it first demonstrates that this process occurs during mycobacterial encounter *in vivo*, implying that Mincle induction by mycobacterial signals could serve to bolster recognition and defense against spread of mycobacteria or secondary challenge by subsequent exposure. In fact, this is exactly what the authors found in their further experiments: when mice were infected twice with BCG separated by 14 days, the inflammatory response to BCG in the airways and in alveolar macrophages strongly increased in a Mincle-dependent fashion. More importantly, the priming infection increased the Mincle-dependence of BCG-growth restriction, showing that the up-regulation of Mincle during the priming period indeed led to better innate control of intruding mycobacteria by alveolar macrophages. Another interesting effect of Mincle-deficiency in this study is the stronger increase in the bacterial burden in the draining lymph nodes and the spleen, compared to the lungs after intratracheal BCG infection (Behler et al., 2012). Dissemination of BCG after airway infection requires time until pulmonary mycobacteria have sufficiently expanded, allowing the quite slow induction of Mincle on alveolar macrophages (and also on other recruited cells, e.g., neutrophils) to generate the higher capacity of BCG recognition and to mount a better response leading to containment of infection at the local site.

Taken together, these recent studies on the role of Mincle in the host response to mycobacterial infection yielded remarkably different results, raising the question for possible reasons that underlie the discrepant findings.

First, the three studies used different strains of *M. tuberculosis* (H37Rv in the Heitmann et al., Erdman in the Lee et al. study) or employed the vaccine strain *M. bovis* BCG (Behler et al.). Although all these strains have abundant TDM in the cell wall, they may differ in the length of mycolic acid chains, modifications and the mixture of other glycolipids present in the cell wall, which may account for the difference in the requirement for Mincle. Of interest, *in vitro* infection experiments of macrophages showed a stronger dependence of the NO production and of G-CSF secretion on Mincle when BCG was used compared to MTB H37Rv (Heitmann et al., 2012), which appears consistent with the larger role of Mincle in BCG vs. MTB H37Rv infection.

The relative amount of TDM present in the mycobacterial cell may also be affected by glycolipid exchange through the action of mycolyltransferases. Increased levels of glucose, as they are encountered by the mycobacteria in the cellular growth environment, can lead to a shift in glycolipid synthesis from TDM to glucose monomycolate (GMM) in an Ag85A-dependent manner (Matsunaga et al., 2008). GMM is a ligand for CD1b in humans (Moody et al., 1997), it has not been formally tested for binding to and activation of Mincle.

In principle, another variable can be introduced by changes in the culture conditions used to grow the mycobacteria before infection. For example, the commonly used inclusion of detergents like Tween in the mycobacterial broth to prevent clumping has been shown to drastically modify the presence of a polysaccharide capsular structure (Sani et al., 2010) and may also wash out TDM from the mycobacterial cell wall. Indeed, the inclusion of Tween in ELISA wash buffers can even remove coated glycolipids from microtiter plate plastic (Julian et al., 2001) and drastically reduce the detection of TDM-specific antibodies from the sera of TB patients (Traunmuller et al., 2005). It is therefore conceivable that inter-strain differences and subtle changes in the culture conditions used can significantly affect the composition of the mycobacterial cell wall and capsule, thereby affecting the interaction with Mincle and other pattern recognition receptors and changing the outcome of infection.

Finally, the hygiene status of mouse colonies can be expected to alter the basal levels of innate immune activation in general and of Mincle expression levels on phagocytes in particular through effects of TLR- and CLR-dependent PAMPs.

## Role of other C-Type lectin receptors in anti-mycobacterial immunity

The adapter protein Card9 is expressed in myeloid cells and was initially identified as crucial for transducing the signal of the C-type lectin receptor Dectin-1 to NFkB activation (Gross et al., 2006). Card9 is down-stream of Syk and becomes phosphorylated by PKC delta (Strasser et al., 2012). Card9 is not only required for Dectin-1 signaling, but is also the central adapter for DAP12- and FcRγ-chain-coupled C-type lectin receptors like Mincle, Dectin-2 and Clec5a (Hara et al., 2007; Yamasaki et al., 2008; Werninghaus et al., 2009). Dorhoi et al. showed that the absence of Card9 renders mice exquisitely sensitive to infection with MTB (Dorhoi et al., 2010). Following aerosol infection, Card9^−/−^ mice develop high mycobacterial burden in the lungs and die within 4–6 weeks. Card9^−/−^ macrophages normally phagocytosed and killed MTB *in vitro*, but were impaired in the production of inflammatory cytokines and chemokines. *In vivo*, Card9^−/−^ mice had a striking increase in neutrophil infiltration in the lung that was linked to tissue destruction and death of the mice by depletion experiments (Dorhoi et al., 2010). This essential role of Card9 in anti-mycobacterial defense, together with the relative lack of a phenotype in Mincle-deficient mice, poses the question which other C-type lectin receptors signaling through Card9 are triggered during MTB infection (see Table 1).

Dectin-1 has been implicated by several labs in the response to mycobacteria, but the nature of the ligand has remained unknown to date. Blocking Dectin-1 with antibodies or laminarin reduced cytokine responses to *M. bovis* BCG or MTB (Yadav and Schorey, 2006; Rothfuchs et al., 2007). In addition, a reduced uptake of *M. abscessus* and impaired ROS production was reported when Dectin-1 was blocked in mouse macrophages (Shin et al., 2008). To test the contribution of Dectin-1-mediated recognition of mycobacteria *in vivo*, Brown and colleagues challenged Dectin-1 KO mice with virulent MTB. The mycobacterial load in the lungs was unexpectedly slightly reduced in Dectin-1 KO mice, but there were no significant differences in lung pathology or adaptive immune responses (Marakalala et al., 2011). Thus, Dectin-1 appears to play a rather redundant role for protection against MTB.

The human C-type lectin DC-SIGN has received considerable attention in the mycobacterial immunity field when it was discovered that binding of mycobacterial Man-LAM to DC-SIGN on human macrophages down-regulates costimulatory molecules and inflammatory responses, presumably *via* induction of IL-10 production (Geijtenbeek et al., 2003). There are several murine homologues of human DC-SIGN in a cluster of seven genes and one pseudogene (Tanne et al., 2009). Based on the binding to glycans with high mannose content and fucose-containing oligosaccharides, mouse SIGNR3 is the best candidate as ortholog for human DC-SIGN. Tanne et al. created knockout mice for Signr1, Signr3 and Signr5, and determined their phenotype in TB infection (Tanne et al., 2009). The mycobacterial load in the lungs of Signr3-deficient mice was clearly increased, whereas knockout mice for Signr1 and Signr5 were indistinguishable from wild-type controls. However, the development of adaptive immune response to TB was not compromised in the absence of SIGNR3 and the mice did not die at higher rates compared to controls of TB. Of note, SIGNR3 was up-regulated on lung phagocytic cells during infection, and the increased mycobacterial load was specific for the lung while no difference between WT and Signr3^−/−^ mice was seen in the spleen. SIGNR3 binding of ManLAM and whole mycobacteria induces production of proinflammatory cytokines through activation of Syk and the kinase Raf1 (Tanne et al., 2009). Although the requirement for Card9 in the activation of macrophages and DC in response to ligation of SIGNR3 has not been formally demonstrated, it appears very likely. Hence, a defect in the signaling of SIGRNR3 may contribute to the strong phenotype of the Card9^−/−^ mice in tuberculosis, although it appears to be essential for anti-mycobacterial resistance only during the early innate phase of infection.

Given the large number of CLR that can be expressed in myeloid cells, additional players must be expected to contribute to recognition of mycobacterial ligands and to macrophage activation and anti-mycobacterial defense via Card9-dependent mechanisms. Another interesting candidate to look at is Dectin-2; of note a Dectin-2-Fc fusion protein binds to the surface of mycobacteria (McGreal et al., 2006). Similar to Mincle, Dectin-2 utilizes the FcRγ chain as adapter molecule. Interestingly, FcRγ chain-deficient mice develop increased mycobacterial burden, enhanced immunpathology and earlier death when challenged with MTB (Maglione et al., 2008). While Chan and colleagues discuss their findings exclusively in the context of FcRγ chain's role as adapter of activating Fc gamma receptors mediating antibody effector function, the dual use of the adapter in recruiting Syk also to several CLR family members could also indicate that the phenotype is at least in part due to a lack of signaling by Mincle, Dectin-2 and other CLRs. Likewise, the activation of the FcRγ-Syk-Card9 signaling cascade by Fc gamma receptors would allow a contribution of antibody-mediated effects to Card9-dependent protection in TB infection, a possibility that has not been explored to date.

### Conflict of interest statement

The author declares that the research was conducted in the absence of any commercial or financial relationships that could be construed as a potential conflict of interest.
